# What Is Commensality? A Critical Discussion of an Expanding Research Field

**DOI:** 10.3390/ijerph18126235

**Published:** 2021-06-09

**Authors:** Håkan Jönsson, Maxime Michaud, Nicklas Neuman

**Affiliations:** 1 Department of Food Technology, Engineering and Nutrition, Lund University, 221 00 Lund, Sweden; hakan.jonsson@food.lth.se; 2 Institut Paul Bocuse Research Center, 69130 Ecully, France; 3 Department of Food Studies, Nutrition and Dietetics, Uppsala University, 751 22 Uppsala, Sweden; nicklas.neuman@ikv.uu.se

**Keywords:** commensality, meal sharing, eating together

## Abstract

Commensality (the act of eating together) is studied in a range of disciplines and often considered important for social communion, order, health and well-being, while simultaneously being understood as in decline (especially the family meal). However, such claims are also contested in various ways. In this paper, we discuss the expanding field of commensality research and critically reflect on the debates surrounding its social functions, including its role in public health. We illuminate the deep social and cultural significance of commensality, through time and space, and conclude that whether or not commensality is the preferred social form of eating for any given individual, it is difficult to escape its sociocultural desirability and idealization. As a cross-cultural phenomenon in both past, present, and future, we suggest that commensality deserves further research. This includes commensality as a research topic in itself and as an entry point to unveil different dimensions of social relations between people, as well as interactions between humans and material objects.

## 1. Introduction

Eating together is one of the most commonly shared practices among human beings, both across space and time. The remains of food around traces of fireplaces, the oldest fireplaces discovered as far back as about 800,000 years ago [1], reveal for how long we can confidently say that human beings have been sharing food. In fact, food sharing is described in evolutionary anthropology as a fundamental part of human evolution, as a means of reciprocal cooperation, which we share with other primates [2,3]. Furthermore, from a bioarchaeological point of view, sharing food and partaking in collective meals have been analyzed as acts that connect the human being as a biological organism to a social person [4]. Understood in this way, eating together makes up a fundamental part of our social nature.

In social sciences and humanities, *commensality* is commonly used as a scientific concept for eating together. However, despite the wide usage of this term, especially in anthropology and sociology, its meaning remains a subject of debate. Does it mean sharing the food? The table? The place? The moment? Maybe the etymology of the term can help us answer this question. The most common stated origin is the word *commensalis*, which appears in medieval Latin. It is formed with the prefix *com-*, used for something shared among several persons, followed by two possible suffixes. One option would be *mensa*, which, here, would designate a table used for food. So, in that sense, commensality is first and foremost a matter of sharing the table and, thus, the place and the central material object. However, the suffix could also be *mensalis*, designating what is on the *mensa*. In this sense, commensality is more about sharing the food itself. Yet another option, never explored as far as we know, would derive the term commensality from the medieval term *commensalia*, which designates the common *symbola* (financial contribution to a meal) left on the *mensa* [5] (p. 447). Commensality could therefore also be more about sharing the cost of a meal than the meal itself.

This etymological digression demonstrates the complexity of the concept. However, if that was not enough, an extra layer of confusion is added when juxtaposing the term with others, such as *conviviality*, which is more about the friendly and enjoyable aspect of being together, or *community*, which designates a group of people sharing characteristics or interests. In this paper, we followed the understanding of commensality as eating together, with or without any specific connection to a table. We use it both as a descriptive term (what people do) and as an analytical term (its social functions). We explore the complexity of commensality by critically reviewing different ways it has been used in social and human sciences, the different conditions it requires, and the different forms it can take. We will provide a number of examples from different epochs and geographical areas. The aim is not to provide an exhaustive overview or “The history of commensality”. The given examples are used to ground a critical discussion of some of the key aspects of commensality research, which will allow us to (1) highlight different approaches and debates within this expanding research field, and (2) point to themes and concepts deserving further attention. Moreover, when we talk about the field as *expanding*, we are not referring to the quantitative increase of publications—which is a fact of almost all scientific fields—but for the diversification of the concept into a wide range of disciplines and research domains.

## 2. Main Directions in Commensality Research in Social and Human Sciences 

The role of shared meals as a symbol of trust (or betrayal), social communion, and reasoned discourse is abundant in philosophical and theological writings, as well as in the arts. Examples are Plato’s *Symposium*, the last supper of Jesus in the New Testament and the Knights of the Round Table. In the early 1900s, foodways as a marker of social classification and cultural grammar grew, especially among anthropologists (e.g., Mauss, Lévi Strauss, and Douglas). Here, the main argument, expressed in different ways, was that the way we engage with food, as in rituals and routines, says something more profoundly about the structure of humanity and cultures as such. Moreover, in *The Structural Transformation of the Public Sphere*, Habermas elaborated on the democratizing function of 18th century London coffee houses [6]. To Habermas, they exemplified a venue of the modern public sphere, with potential for organization, social participation, and democratic discourse. The product as such, the coffee, is not in focus, however, but rather the potential inherent in the social occasion made possible through drinking it with others in public. A history of commensality research may begin with Georg Simmel’s “Sociology of the Meal” [7], in which he argues for the meal as a site in which the cultural and the natural coincide. The meal is, at the same time, an individualistic act and one that is socially shared and regulated. In fact, the very naturalness of eating, something every human being must do, is precisely what makes it so fundamentally social to Simmel.

In the late 1970s, Claude Fischler laid the first cornerstone to a still ongoing systematic theorization of the social function of sharing meals. Fischler [8] claimed that, in Western societies, “meals are being increasingly eroded by or reduced to snacks. Eating is becoming less of a social, and more of a strictly individual, practice.” In such societies, he further suggested, shared rules and norms associated with food (what he calls gastronomy) were eroding. He called this gastro-anomie, a concept rooted in Durkheim’s theory of anomie—a societal condition of guiding norms in decline, in which the individual’s belief system is incongruent with that of the community. A particular *gastro*-anomie, then, is when the community’s gastronomy is not in line with the individual’s. This, Fischler argued, has negative effects such as “nutritional disorders” [8], by which he seems to mean both psychological anxieties and physiological health effects. It is, in other words, an argument about how food becomes a means of social integration, whereby the corrosion of gastronomy (rules and norms associated with food)—most clearly manifested in the (supposed) decline of shared meals—can be traced in society’s pathologies.

Fischler would then set forth a research program of investigating the seemingly universal traits of food and sociality, as well as the culture-specific differences, for many years. In 2011, many of his arguments from years of scholarship culminated in a paper that places commensality at the very center of human culture [9]. He even mentions the differences in prevalence of obesity and associated disorders in France and Italy compared to the United States, suggesting that the diverse cultures of commensality may explain why these differences exist. Due to a reduction of food to mere nutrients and the emphasis on individual choice, the argument goes, eating lacks social regulation in countries such as the United States. Such regulation, through norms of appropriate behavior, is then suggested to exert social control “upon eating behaviour and plays an important part in setting amounts consumed by individuals” [9].

Similar to Fischler, Falk [10] argued that instead of viewing social solidarity as primarily grounded in shared values, as has been a common sociological understanding, there is a more “primitive” origin of solidarity in our eating communities. As such, through rituals of food and eating, societies are formed and upheld. Falk is clearly distinguished from Fischler in his emphasis on the body, however. This is particularly evident in the role he ascribes to the mouth, which works bidirectionally as both incorporating the food of the community into one’s body while “being eaten into the community” [10] Still, the conclusions are very similar to Fischler’s in that an individualization and destructuration of modern eating results in loss of social solidarity. In fact, Falk goes as far as stating that the eating community “as a structuring principle of social life” is collapsing in the modern condition, and that this is manifested in the marginalization of the shared meal [10] (p. 29). Such decline of the meal, he further argues, encourages snacking and “other modes of concrete oral-ingestive activity which concerns substances that are not considered to be food” or nutrition [10].

Moreover, a classic text of the social sciences that is conspicuously absent in the commensality literature is Putnam’s *Bowling Alone* [11], a book that includes several references to eating with others. More precisely, analyzing the decline of social capital in the United States through a suggested decrease in civil engagement, he provides data both on shared meals with families and friends as well as dining out. People agreeing with a statement about having family meals each night had declined and many had their meals in front of the TV, Putnam writes. Moreover, he describes a decline in full-service restaurants, bars and luncheonettes at the same time as “the per capita number of fast-food outlets, those ‘personal refueling stations’ of modern society, doubled” [11]. What such evidence suggests to Putnam is that the time and place devoted to conversations and sociability—where social bonds are strengthened—are being substituted for places where people “grab a bite and run rather than sit a while and chat” [11] (p. 102). Thus, there are traces in Putnam’s writing of both Habermas’ argument about public discourse, as well as Fischler’s and Falk’s on social communion, although none of them are cited.

As of Putnam’s resemblance to Fischler, it is quite clear that they share a more or less functionalist view of social engagement. However, despite the fact that Fischler relies on a broader concept of “individualization” of society, he nevertheless sees eating as a particularly central aspect, while Putnam uses it as one of many examples of something bigger (a general decline in civil engagement). For Falk, eating communities reflect the foundation of human civilization, as the form of social community in our pre-modern society. This idea may also support Fischler’s and Putnam’s hypotheses and, additionally, resonates with the aforementioned evolutionary arguments about food sharing, in which the reciprocal sharing of food has been highlighted as fundamental to human cooperation [2,3]. Furthermore, the idea is in line with many qualitative studies showing that commensality is seen and presented as important for social cohesion and cultural identity [12,13,14,15].

That said, scholars are not in complete agreement with these interpretations of shared meals in decline or their (causally) negative implications. To begin with, another French scholar, Grignon, approached the question differently [16]. This is made clear already in the first paragraph of his text on commensality and social morphology: “Consuming food and drinks together may no doubt activate and tighten internal solidarity; but it happens because commensality first allows the limits of the group to be redrawn, its internal hierarchies to be restored and if necessary to be redefined.” [16]. In other words, the social bonds formed and strengthened by commensality also functions as social demarcation and hierarchical distinction. He discusses different ways in which commensality exists, and thus their manifold social effects, by formulating a typology of three binary classifications: domestic and institutional, everyday and exceptional, segregative and transgressive.

Domestic commensality refers to the home, which may provide increased closeness among family members and friends, but also the manifestation of power asymmetries based on gender or other social positions (e.g., the master and the servant). Institutional commensality is hierarchical by its very nature “and dominated by the rule of the institution” [16]. Rank, pupil-teacher hierarchies, and other kinds of formal distinctions are at the very core of such commensality. This is not to say that institutional commensality is inherently “oppressive”, only that it is based on principles very different from those expressed by scholars such as Fischler. As of everyday and exceptional commensality, the difference lies in its degree of frequency, demand for planning, and external constraints (work schedules, size of the household, etc.). In other words, the everyday/exceptional binary means a continuum of required efforts and the social codes involved in its execution. The social functions and effects are likely to differ.

In what Grignon calls “segregative commensality”, eating and drinking is viewed as “a way to set up or to restore the group by closing it, a way to assert or to strengthen a ‘We’ by pointing out and rejecting, as symbols of otherness, the ‘not We’, strangers, rivals, enemies, superiors or inferiors” [16]. Thus, the social bonding here is just as much, or even more, a social distancing. An example of this, to Grignon, is the Indian caste regime, where the distinction of people is not only manifested through segregation, but also through the types of food eaten. We might also add the situation in Medieval Europe, where the three main religious communities differed in regards to what was considered proper food, which spurred long and heated debates among scholars of all religions about whether it was possible for Jews, Christians and Muslims to share a meal [17].

Transgressive commensality, then, surpasses such distinctions. As in Habermas’ idea of the coffee house, a variety of people may deliberate, using reason to understand each other better and transcend what seemed to be social conflict. Still, Grignon is not completely optimistic here either, pointing to the remaining power asymmetries involved in, for example, “the ‘invitation au château’ (invitation to the manor) or the political leader having lunch with the workers at the factory restaurant” [16].

One should not exaggerate neither the optimistic view of shared meals from Fischler/Putnam nor the critical aspects from Grignon. All express important nuances that may be lost in a brief summary as this one. However, the tendencies in one or the other direction are clear and can be classified in accordance with one of social theory’s classic distinctions: the one between consensus and conflict—that is the analytical priority of social order through shared norms and actions and social difference through the divergence, rooted in social stratification, of group interests.

Another criticism is of a more empirical nature, namely Murcott’s discussions about the claimed decline of the family meal and, more importantly, whether or not one can draw conclusions about beneficial or detrimental effects of such a decline [18,19]. First, Murcott points to the low quality of the evidence in support of this proposition. Counter to the proposition’s claim, for example, is a study based on over 400 life-history interviews from families of Edwardian England, suggesting that family meals were not as common as some—who lament its decline—might think [20]. However, even more troublesome to Murcott, in line with Short [21], are the attempts to draw causal inference from such data to other social problems. Moreover, she distinguishes between the family meal as an idea and an actual practice, referring both to her own as well as other scholars’ work in the past that, in different ways, challenge the narrative of the social decadence due to declining family meals. This argument is closely related to a discussion of Wilk about what he calls a “U.S. National Political Agenda” of family meals [22]. Wilk here distinguishes between the normative and the performative aspects of the family meal, meaning the discrepancy between the family meal as a romanticized ideal associated with “intimacy, integration, interaction, sociability, connection, foundation of society, togetherness, harmony, love, and nurture” [22] and its actual practice that can involve conflict, subordination, and coercion. Moreover, Wilk does not only challenge the normativity of family meals, but also criticizes his fellow anthropologists of reproducing this idea, despite anthropological observations suggesting both that the nuclear family is not always the main unit of commensality and that commensality is not a universally preferred form of eating. He does, however, agree that eating seems to be a universal form of social life in all societies that have been studied. 

Murcott also discusses matters of social stratification and divisions of labor, again echoing the critical analyses of Grignon and Wilk, in that the family meal is a middle-class ideal that usually builds on a gendered division of domestic foodwork. In sum, Murcott reviews a decade-long academic debate about family meals and commensality in general—one that is far from any consensus, despite commonsensical claims about its almost magical properties for our health and well-being, especially for children (the actual evidence is something we return to further on). Murcott’s argument was then furthered by scholars such as Parsons, who directed attention to the distinctions of class and gender involved in taken-for-granted notions of “healthy” family foodways, such as sitting around a table eating organic food cooked “from scratch” [23]. Such notions, Parsons argues, create aspirational models of functioning families that may demonize necessity-based choices of convenience food or other dimensions of a meal that signals (lack of) cultural and symbolic capital. “The sharing of everyday family foodways” she writes, “centred on inculcating cultural values through the sharing of food, culture and experiences. This is not just about economic capital … but also concerns the display of cultural capital around everyday foodways.” [23]. Similarly, in a recent study from the United States, Bowen, Brenton, and Elliott show how an incapability to provide ideal family meals can be a source of guilt and shame to mothers struggling with food insecurity [24]. This lends further empirical support to the phenomenon of commensality—particularly the family meal—being idealized and considered worthwhile, but also, and precisely *because of* its culturally cherished position, being a marker of moral failure when reality does not reflect the ideal.

In sum, some of the research cited above share the view that commensality is declining and that this is a challenge for contemporary (Western) societies. In recent decades, however, a new generation of scholars have continuously contributed with empirical studies, showing a more multi-faceted view of commensality. The more recent directions invite us to turn away from the idea of a taken-for-granted decline of commensality into an exploration of its contemporary evolution. Below, we discuss dimensions to consider when studying commensality (concrete dimensions), and then analyze contemporary aspects and potential future research trajectories.

## 3. Concrete Dimensions of Commensality

### 3.1. Material Aspects of Commensality

As described above, the word commensality can itself, depending on the definition, imply a material aspect—that of the table (*mensa*). The material aspect of commensality further includes the utensils of the meal situation and its place. Different ways of organizing communal eating is to some extent dependent on the material infrastructure involved: stones around the campfire and wooden sticks to hold the roast, pottery for cooking, preserving and serving of the meals, and roman divans for eating while lying down. These are some historical examples of how materiality is important for commensality.

A dominant view in most cultures allowing alcoholic drinks as part of a meal, is that they should be taken together with the food. In some countries, such as Sweden from 1917 to 1955, it was even prohibited to serve alcohol if this was not accompanied with a cooked meal [25]. During Greek antiquity, this would have been a very odd practice, since food and drinks were dictated to be separated. First, the meal was taken; then the symposium, devoted to drinking, could begin [26]. Needless to say, this affected the interaction between people and material objects during the meal. Holding a glass or jug, or navigating between bowls, plates, platters, knives, spoons, and other objects created different spaces of commensality. Furthermore, material objects can function as mediators of power relations. In notes on habits of 18th century rural populations in Sweden, it was noted that the woman of the house did not have a chair at the table. She should stand up during the meal, being ready to pick anything missing at the table [27]. The long table, with the throne or high settle at the short end and people sitting according to their rank further down the table is another example. In contrast to this, the round table in the tales of King Arthur was deliberately designed to manifest equality. 

Hierarchies manifested through materiality is not a thing of the past, however. Returning to Wilk [22], he also discusses power relations at the table by pointing to the importance of the small things. Who is allowed to hold the pot and casseroles to serve others or themselves? A mother holding the pot or a casserole, serving members of the family, may be seen as a simple act of caring, but deciding who will be given what and in which order is also an effective way to maintain hierarchies around the dinner table. In fine dining, moreover, the competences of navigating in the landscape of objects have been taken to the next level. Anecdotally, many people felt uncomfortable the first time they sat down at a fancy dinner table, looking at all the glasses and cutlery. What should be used for which course? Table manners include a lot of silent knowledge, knowledge dependent upon the cultural capital brought to the table. The materiality of meals thus carries with it social connotations, and may induce a sense of community, but also evoke sentiments of loneliness, and mark distinctions between us and them. 

In order to break up with the strict regulations of fine dining and table manners of the bourgeoisie, the growing middle class of the counterculture era searched for alternatives. The popularity of the fondue pot in some circles in the 1970s is an example of a willingness to reinstall a sense of commensality in festive meals. However, it is not only in fine dining that the social codes involved around food are manifested, both symbolically and materially. As demonstrated by Julier [28], this is even the case for the American potluck, a seemingly democratized form of dinner event in which the guest (in theory) brings whatever he or she finds reasonable. Viewing the potluck as completely democratized, without social expectations or means of cultural distinction is, as Julier shows, deceitful. In fact, a great deal of expectations on guests are silently assumed. What is at first sight a banal choice of bringing a bag of potato chips may in fact be a clear marker of cultural capital, symbolizing “bad taste”, laziness, cheapness, or simply a lack of what Bourdieu called a practical sense of “the game” [29]. 

As we have seen, the material dimensions of commensality have changed in history, reflecting as well as being active parts of cultural change. The notion that the materiality has changed should not, however, be overestimated. On the contrary, there is some striking continuity, not least when it comes to the most common goods for meals. After the introduction of the fork in the latter phase of the medieval epoch (elegantly analyzed by Norbert Elias [30]), not much has happened in the Western hemisphere. A spoon, knife, fork, plate, glass, and jug was as common to a late medieval middle-class person as it is today. Changes in the design and material of the goods have taken place from new production methods and trends. China has replaced wood as material for plates, apart from where images of a rustic rurality is wanted. While spoons have shrunk during the last hundred years or so, the wine glasses and the coffee cups have grown [31]. 

Overall, we would argue that the material design of eating has undergone less dramatic changes than other domestic activities, such as cleaning and washing. That being said, there is certainly room for variation even if the same material objects are used. The orchestration of a restaurant meal has had its different ways of serving and eating. *Service à la française*, meaning that several courses are brought out simultaneously, was replaced by *service à la russe* in formal dining in many European countries in the 19th century. The new style meant that courses were brought to the table sequentially, and the food being portioned on the plate by servants at a sideboard before being given to the diner. In the nouvelle cuisine era, the *service à l’américaine* or *service à l’assiette*, meaning that the food is put on the plate by the chefs in the kitchen, became the norm. Such examples have created distinctly different manners of serving, as well as the types of food being served. 

### 3.2. The Participants and Their Relationships

Like many rituals, commensality involves the creation of a particular space-time through material settings described above. However, it also involves some material or immaterial dimensions directly linked to the participants themselves, such as the respect of a certain number of explicit or implicit rules. These can regulate which clothes are acceptable or unacceptable to wear, like the injunction to remove the hat while eating in many Christian societies, or the obligation to wear a clean pagne for the Wayãpi Indians of French Guiana invited to drink beer during great parties [32]. Less material, the content of the oral exchanges between participants is also highly regulated. In different societies, the questions of who is allowed to talk and the acceptable topics of conversation have been regulated with more or less rigid sanctions. To “let the food shut the mouth” was imprinted in generations of people in Sweden, for example [27]. In many hierarchical (patriarchal) settings, it was the head of the family who was in charge of the conversations, while the others were not allowed to talk unless explicitly encouraged. In some situations, talking while eating is completely forbidden, for example for monks who use signs when they need something specific, such as asking for bread or butter [33], or in many other cultures where the talking is done mainly *before* eating [34]. However, more generally, even if talking is authorized, the type of subjects that can be broached is restricted. Forbidden topics are generally those which can generate disgust, discomfort or conflict, at least since the late Middle Ages [35]. In France, as in many other European countries, it is also considered rude to do something else than talking with others while eating; especially doing something alone, such as reading newspaper or using the phone [36]. Consequently, the importance of smartphones in everyday life of the last decade has become a challenge for many parents as the question of whether or not to allow it at the family table can become a source of conflict [37]. 

Beyond the creation of commensality, the respect of these rules generates a sense of belonging to the same community. In Western countries, having someone over for dinner is putting your social and cultural capital at stake. The higher the social position of the guest is, the more formal the dinner setting will be, both regarding material aspects and the rules guests have to follow. Vice versa, the closer the guests are (in the terms of kinship or social category), the lesser strictness of the rules—eating a pizza by hand while watching TV is restricted to people of close social proximity. However, the codification can sometimes be more complex, as the trends of the “Luncheon on the Grass” in the French bourgeoisie of the late nineteenth century shows. Even if these lunches were also highly regulated, changing the rules by authorizing eating on the grass aims to create a festive atmosphere and a special moment (this aspect still exists in the form of the contemporary picnic). This example underlines that varying rules and norms can be a way to change the kinds of relationships created with other eaters. More generally, in many cultures the codification is interlinked with the social or generational status of participants, which makes the shared meal a good moment for anthropologists to observe the social position of the members of the society [38]. 

However, some intangible participants can also be part of this eating community. Many religious rituals involve food sharing among participants, such as the Eucharist in the Catholic mass. Sometimes the food is also directly shared with invisible entities, as in ancient Mesopotamia, where the gods had their own places at the table [39]. The practice of libation, for example, sometimes involves water or alcohol drunk by the offering giver and then symbolically “given” to the entities (gods, spirits, ancestors) by versing on a specific spot, statue or natural element. This is the case in some voodoo rituals in south Benin, where alcohol is “shared” with the *vodun*. In Romania, for Easter, food is traditionally shared with the dead ancestors, in the form of the *pomana* [40]. However, these kinds of sharing can also be part of everyday eating or drinking practices. In West Africa, especially with alcohol, it is common to verse on the floor a part of the drink for the spirits, before drinking it, which Joly has described among the Dogon for millet bear drinking [41]. The same practice is largely widespread around the world, for instance among the Laotians in the sharing of the *laô laô* (rice alcohol) [42]. These examples underline the fact that some invisible and metaphysical entities can also be thought of as partaking in the everyday meal. 

### 3.3. Orchestrating Commensality

As shown thus far, commensality does not emerge arbitrarily as soon as people decide to eat and drink. In different contexts, both at a commercial and a more personal level, some have taken on the task to orchestrate commensality. In the domestic setting, the role of the maid, the housewife or the butler has to some extent been dedicated to the orchestration of commensality. The same holds for large sections of the modern food service industry. To treat the members of the meal equally but individually at the same time is an important skill that any waiter or waitress knows will result in tipping. Working to avoid frictions, such as long waiting time, uncomfortable chairs, a too loud or too silent environment, and many more things, can be added to the commensal situation. Moreover, as any person skilled in hosting shared meals knows, successfully creating commensality is a very difficult act of balancing with many potential pitfalls—including welcoming the participants of the meal, securing drink and food supply in the expected time, managing the conversation, and finally breaking up from the meal in a good mood. Many small actions will influence the success of the commensality. 

However, it can be noted that the orchestration of commensality is rarely communicated to the participants. Fine dining restaurants seldom market their offer of a social space but prefer to highlight the sensory and performative aspects of the food and drink. The use of the word hospitality industry and not commensality industry is perhaps telling regarding how the sector views the role of its establishments. When it comes to the offering of commensality, it seems as if the main experts are located in the low- or mid-range establishments of the food service sector; the small inns, taverns and bistros attract clients that are searching for a commensality experience rather than a gastronomic one. This aspect was well described by Chauffier, in a chapter about a French family meal in a fast-food restaurant [43]. She points out that the parents choose fast-food restaurants based on the low normative and behavioral constraints it involves, thus facilitating the relationship with the children. She underlines to what extent it represents a change in the use of public meals, which are traditionally more regulated than the domestic ones, and an occasion to demonstrate the standing of the family and the quality of the education of the kids. On the contrary, in fast-food, everything is designed to limit the constraints and allow the children a lot of freedom, such as eating with the hands or standing up and playing in the middle of the meal. Some restaurants are especially designed to create social relationships, like the ones created by the association *Les Petites Cantines* in France. They offer customers to be involved in the cooking if they want, and isolated tables have been replaced by long tables to force social exchange between them. This represents an interesting historical come-back, as the common large table was the original way of eating in the *auberge*, before the introduction of separated tables around 1760 gave birth to the modern *restaurant* [44]. 

In a period where single households become more common, it seems fair to argue that the commensality offerings of the food service industry are becoming more important. It remains to be seen if this insight will lead to new marketing strategies for some parts of the food service industry. What is more, a trend among foodies—people with a passionate culinary interest—has emerged about eating out alone. If this will grow or fade is impossible to say at the moment. However, as argued by Koponen and Mustonen, drawing on interviews with 12 food and restaurant enthusiasts in Finland who advocated solo dining, this practice is considered an aesthetic improvement—letting the diner focus entirely on the food itself—as well as one that still involves a great deal of sociability [45]. The authors argue, perhaps counterintuitively, that solo dining constitutes a culture of togetherness through a social connection between people, staff, and customers (including online interaction), who share gourmet interests. “The aesthetically oriented solo diner finds pleasure in ‘consuming’ the traditional commensality produced by the other tribe members at a restaurant”, they write, “but is freed from the burden of actively participating in such production. S/he is simultaneously alone and with others, striving for a balance between togetherness and aesthetic immersion” [45]. If we assume that Koponen and Mustonen are on to something that has further transferability, then commensality in the public sphere may require redefinition, at least among a small subculture of enthusiasts. This trend and its potential implications for the future brings us to the next section.

## 4. Current and Future Trends in Commensality

After digging into the many different aspects of commensality, a fair question to ask would be what the current situation of commensality in the world really looks like? The answers are manifold and dependent on perspective. 

### 4.1. A Decline of Commensality?

There are certainly many doom and gloom advocates, claiming that we live in a time of commensality crisis. Individualized eating, decline of family meals, lack of shared norms in everything from table manners to what is considered to be edible substances (e.g., meat) are phenomena that, to the extent that they are true, could indeed discourage commensality.

However, there are tendencies that challenge the idea of the decline of commensality. The food service industry is rapidly growing, with a considerable share of food eaten out of home [46]. Even though the statistics do not make it possible to differentiate between shared meals and single people eating out alone, one need only to take a look at city centers at an average night throughout the world to see that there is ongoing commensality in bars, cafés, pubs, and restaurants. According to a recent book by Warde, Paddock, and Whillans [47] the frequency of meals eaten out in England seems to have been rather stable from 1995 to 2015, at least in London, Preston, and Bristol where the study was conducted. However, while it seems as if people may not eat out to a considerably higher extent, the way people do it has changed. Warde and co-authors argue that eating out has gone through processes of familiarization, informalization, and diversification. Once something unusual and out of the ordinary, eating out has now become a day-to-day activity, with less formality attached to it (e.g., regarding dress and manners), while the types of cuisines, events, and establishments to try out have become much broader. This has implications for commensality—in terms of with whom the public meals are shared, new social formations of hierarchy and distinction, and the individualization of things to eat (e.g., the difference between eating at one small-menu á la carte restaurant compared to a food court). 

Moreover, social scientists have shown how shared eating across time and space in the domestic setting gives us insights into larger patterns of social transformation and synchronization (i.e., that people eat on a regular schedule that may or may not harmonize with the overall pattern within a society) [48,49,50,51,52,53,54,55]. Moreover, as it turns out, commensality does not seem to be particularly “threatened”. In the Nordic countries, regarded as highly individualistic and with progressive family policies, a study found that a little more than one third of the meals were reported to be eaten alone, less common than family meals (approximately 42–46%, depending on the country) [55]. Some data, for example in the United Kingdom [51], Belgium [49], and South Korea [53], suggest a slight decline in some forms of commensality, but the general picture seems to be that even though commensality changes—both practically and symbolically—it continues to be socially valued and desirable. Examples on the contrary exist, where people prefer eating in solitude, but one must distinguish between individuals and subgroups who may choose other paths and broader social erosion of the sorts that Fischler has alluded to. As mentioned above, the threatened the family meal, for example, is more a rhetorical figure than an empirically-based fact. There are clearly several issues remaining concerning how cultural capital is associated with how commensality is expressed, but this is a different matter than that of actual prevalence over time or between cultures and nations.

### 4.2. Specific Diets and Commensality 

In recent years, the growth of more or less documented intolerances to certain food (gluten, lactose, soy) and the connected boom in “fad” diets (low carb, paleo, etc.) is said to have posed further challenges to commensality. In Sweden, staff at conference hotels, school diners, and many more places claim to have a hard time trying to comply with all requests for special diets [56]. Fischler, in a book he edited on selective eating, defends the same idea that special diets are challenging commensality, going as far as subtitling his book, “Will we still be eating together tomorrow?” [57]. However, again, this depends on the perspective. In a study of cultural aspects of coeliac disease, Cridland [58] noted that the boom in “free from” products actually encouraged commensality from the coeliac community’s point of view. For the first time, they could share the same meals as other members of the family and together with friends. When gluten-free bread became readily available, less expensive, and (fairly) tasty, whole families could live on gluten-free diets. Whether a specific diet creates exclusion or inclusion thus depends on the situation. Moreover, as already mentioned, for some Finnish food enthusiasts solo dining can still carry the social functions we ascribe to conventional commensality [45].

### 4.3. Digital Communities

Another aspect of the sharing of food are the emerging digital platforms. Online eating communities are formed around veganism, barbecue techniques, edible wild plants, dashi, naturally fermented wines, and more. These eating communities may not be commensal in the strict definition of the word, but food communities that do not require geographical proximity may also create similar senses of belonging through food and drink as the shared meal may do [59]. A cultural phenomenon related to this is *mukbang*, originating in South Korea, but now with global popularity, in which social media influencers eat (often great amounts of) food in front of the camera while interacting with the audience [60].

### 4.4. Cooking Together

Finally, there are some interesting tendencies that while eating may be individualized, cooking may actually become more of a joint work. Frances Short [21] noticed that “Cooks Want to Cook Alone” (p. 38, capitalization in original). Moreover, there are indeed many anecdotes of how the cook (usually the mother) with more or less brutal force made sure to keep everyone else away from cooking. However, the emergence of cooking as a lifestyle and leisure [61] may have also transformed it into a more sociable practice. A study among men in Sweden found that, for some, cooking with others (friends, partners, children) was considered as enjoyable as sharing the food [62], an extended sociality from eating to cooking that has also been observed in Belgium [63], England [47], and among older people in Denmark [64]. A return to earlier practices of communal cooking and baking, common in most villages way into the 20th century, may therefore compensate for any potential decline of commensality [65]. 

## 5. Questioning Commensality

### 5.1. Chosen vs. Suffered Commensality

Very often, commensality is presented as positive, generating sociability and conviviality. Conversely, eating alone is associated with isolation and loneliness. It is, for example, the case of the elderly in France, for whom it is considered—either in the media or in public policies—that eating alone goes along with being lonely and undernourished [66]. However, commensality can take on negative dimensions, particularly when it involves an injunction or even coercion. This was discussed by Watson in a chapter devoted to the public mess halls set up by Mao’s China from 1958 to 1960 [67]. At that time, the authorities banned domestic kitchens in favor of communal dining areas. One of the objectives was to break up the family unit, which indicates that the major role of commensality in family cohesion had clearly been identified by the Chinese authorities. Watson reports the particularly negative testimonies of witnesses at the time, who have very critical memories of this “coercive commensality”. If this type of attempt to create cohesion that goes beyond the family by eating meals together exists in many community groups, Watson insists on the specificity of the fact that the Chinese at the time had no choice and could not avoid it. 

This example, admittedly extreme, highlights a difference between chosen commensality and suffered commensality, which strongly conditions the commensal quality. At a personal level, most people can probably recall at least one experience of a shared meal where one would have preferred to be elsewhere, even alone. A study based on interviews with people from Oregon, United States, most of whom had experienced socioeconomic hardship, confirmed how food in the family (including the meal) could be remembered as a source of pleasure, care, and love, but also revealed food’s association with family adversities [68]. The latter concerned aspects such as abusive behavior and dominance, and the authors note that such experiences of family food must be considered as well when family meals are discussed from a public health perspective. Particularly in connection to families struggling with socioeconomic hardship. 

Beyond this, the importance of choice in commensality questions other situations, particularly with regard to what Grignon called “Institutional Commensality” (cf. above). In school settings, commensality was understood as valuable for children’s mealtime interaction in England [69], while a study from Sweden found that the meals were both considered to be a an appreciated arena for sociability as well as a space for feelings of exclusion and loneliness [70]. A Danish paper further argued that the social functions involved in commensality do not necessitate that the same food is eaten, since the children interacted in similar ways around their lunch packs brought from home [71]. In fact, the different lunch packs even opened up for other forms of symbolic exchange, due to the potentials of sharing of food as gifts. Additionally, the authors highlight that when the same food was served to all pupils (as it was during one stage of the study), children could be subject to social exclusion when they avoided certain foods that are conventional in Danish food patterns (e.g., because of religious reasons). 

School meals do not make up all of a child’s meals, however, and there may be a great deal of flexibility in how long one must sit, with whom, a gradient of freedom depending on the age of the pupils, and so forth. Other institutions are different. For example, based on a case study with English men in prison, it was argued that through lunches eaten externally to the prison environment, commensality made inmates’ interaction with people outside possible, making the inmates feel more accepted and “normal” [72]. In retirement homes, furthermore, the sharing of meals between residents is often considered a means to reduce social isolation, and is generally seen as positive. However, in an ethnographic study carried out in several French nursing homes, Guérin showed that these meals were far from exclusively convivial [73]. Some residents clearly expressed the desire to be able to choose to eat alone or with a few selected residents rather than systematically in the common mess hall. Beyond that, she highlights the way in which the caregivers in charge of the meal tried by various means to simulate a rather artificial conviviality. As yet another example, a Swedish study conducted at homes for people with mild intellectual disability showed that sometimes eating alone was the preferred choice [74]. No doubt, commensality was oftentimes enjoyed and the authors discuss the positive aspects of commensality for creating and strengthening social relationships. However, there were nevertheless examples of the opposite, for example when table neighbors were anxious or lost their temper, or when some residents simply did not feel like socializing, and this included times when commensality was more or less forced. These examples highlight the risk of considering, in public policies, commensality one-sidedly as a “good thing”, even if it means imposing it while forgetting the importance of the voluntary dimension in the pleasant character of commensality. We will return to this point in our concluding discussion below.

### 5.2. Commensality for Public Health?

In recent years, commensality—the family meal in particular—also received public health attention. Most of the research in public health focuses on demonstrating the general benefits of commensality, both in terms of physical health and psychosocial well-being (or even a combination of the two) [75]. They often provide data suggesting beneficial health effects of frequent family meals for children and adolescents, such as psychosocial health, nutritional health, and other benefits [76,77,78]. Commensality has furthermore been suggested as one key social influence on eating behavior in later life, with potential benefits both in terms of culinary pleasure and nutritional status [79]. Family meals have been linked to higher consumption of key nutrients, including fruits and vegetables, and a lower consumption of sweets and soft drinks [80,81]. Studies from South Korea have also demonstrated that people who ate fewer meals together had poorer mental health [82] and that eating together is associated with better life satisfaction [53]. A Brazilian study further showed how increased pleasure in commensality may be a part of successful health intervention among women with obesity [83]. Public health research also covers attempts for combatting the lack of commensality. Finding new roads to commensality in widowhood [84], or among young adults have been studied in a Danish context [85], for example. Based on studies demonstrating the positive health effects of commensality, eating together has even made its way into national nutrition policy in some countries [52,86,87], while civil initiatives are strongly promoting family meals [88,89]. 

Combining the health-related arguments above with social-scientific arguments about shared meals’ important function for accumulation of social capital and social regulation, then surely the question must be settled? Not really. To begin with, the family meal studies are almost exclusively observational, mostly cross-sectional, meaning that we should be careful about making claims about causal effects of the meals themselves or whether they may perhaps only be markers of other beneficial circumstances (i.e., socioeconomic stability, low levels of family conflict, etc.) or a result of genetic confounding. A recent systematic review brought out the lack of evidence to determine the causal relationship between family meals and health and wellbeing, pointing especially the lack of intervention studies [90]. This has also been highlighted by a recent narrative review [91]. 

Studies on older people vary greatly, and the population is extremely heterogeneous. Moreover, as Björnwall et al. pointed out, studies of community-living older people have the same problems of causality as the studies on family meals [92]. It does indeed seem to be the case that many enjoy shared eating, and several studies do suggest that it has benefits. However, causality remains unclear, especially since commensality as such is measured in heterogeneous ways. Moreover, the social influence on food intake from eating with others usually leads to a short-term increase or decrease in caloric intake depending on how the intake was modeled [93] or which types of informational norms about food intake that participants were exposed to [94]. One recent meta-analysis of naturalistic and experimental studies even found clear evidence for increased intake when people ate with friends and family compared to eating alone, but no effect for eating with strangers [95]. If such results can be extrapolated outside the study settings, then this indicates that commensality with family and friends could even be negative for weight maintenance. Perhaps a positive effect in some circumstances, such as older persons with reduced appetites, but not for the general public. Fischler’s [9] argument is that short-term effects of modeled higher intakes in controlled environments fade out due to the social regulation that the meal exerts in everyday life. This is an interesting argument, but the evidence is uncertain.

There is little doubt that commensality matters to many; across age groups, social classes, cultures, societies, and historical time periods. As such, it probably matters for the well-being of numerous individuals and groups—in strengthening social relationships, expressing cultural identity, and partaking in civil communities. However, we are less convinced about general guidelines to whole populations with suggestions about specific health effects (e.g., consumption of healthier foods or prevention of overweight). Guidelines to a population provide normative direction, and this has to be weighed against the strength of the evidence, probability of certain outcomes, and their severity. Some people choose to avoid commensality, for a host of good reasons and in a variety of different situations. Yet official guidelines would suggest that they are making decisions detrimental to their health, a suggestion that would be based on weaker evidence than what is normally required for claims about the connection between nutrients and non-communicable diseases, for example. 

The public health potential of commensality thus remains an open question, calling for further empirical studies from a variety of angles, disciplines, and geographical and cultural contexts. Moreover, we agree with Scander et al. [96,97] that learning more about commensality’s role in food and health requires reliable ways of *assessing* eating together, for example, as part of studies on dietary intake and meal timing.

## 6. Conclusions

In this paper, we attempted to show the deep social and cultural significance of commensality through time and space. Whether or not commensality is the preferred social form of eating for any given individual, it is difficult to escape its sociocultural desirability and idealization. We hope that this discussion shows that the complexity of commensality should make both researchers and public health professionals cautious with simplistic statements about the values of shared meals. However, even more importantly, we wanted to present commensality as a research field with many paths to explore in the future. Although this research field has expanded in the last decades, there are still open questions for future generations of food researchers. We would claim that commensality, as a cross-cultural phenomenon in past, present, and future, is well designated to be a topic for further research. Both in itself and as an entry point to unveil different dimensions of social relations between humans and material objects. 

We especially suggest to go deeper into the contemporary evolutions and functions of commensality. In terms of public health, we suggest that methods be developed to identify causality; for whom and in which circumstances can commensality be beneficial or detrimental, both in terms of physical health and psychosocial well-being? Another path is the effects of the ongoing changes in public and private spaces. What effects on commensality can be derived from the combination of an increase of single households and a growing food service industry? Yet, another theme is the effects of global mobility of both people and goods. Numerous studies have shown how meals can provide a sense of community and of conflict, joy and fear, belonging and alienation. More ethnographic accounts of meals in multicultural settings in the “global village” would be relevant, to shed further light on how these frictions may shape future forms of commensality.

As a final reflection, it is hard to overlook the historical context in which this article was written: the COVID-19 pandemic. The restrictions imposed had immediate effects on the sharing of meals. School canteens closed down or were forced to re-organize, older people were denied the option of sharing meals with family members and friends, and the food service sector faced a previously unimagined recession. At the same time, confined families had little choice but to eat their meals together during the weeks, which, as we have discussed above, does not always imply positive experiences. The long-term consequences of the pandemic in relation to commensality is a topic that deserves attention. Will we gravitate to normality as soon as we can or will some transformations in our meal routines stabilize, and how will it develop in tandem with new patterns of migration, urbanization, and environmental effects on the food system? There are many possibilities of upcoming research questions. And regardless of which directions commensality research will take, the point is that although humans may have been a commensal species from the very beginning, the concept, as well as the practices, are constantly open for change as new generations face new realities.

## Data Availability

Not applicable.
